# Effectiveness of Bacille Calmette-Guerin vaccination policies in reducing infection and mortality of COVID-19: a systematic review

**DOI:** 10.1186/s41256-022-00275-x

**Published:** 2022-11-07

**Authors:** Joseph Christian Obnial, Mystie Suzuki, Catherine Joy Escuadra, Janine Trixia Austria, Ma. Jamaica Monique Ponce, Elaine Cunanan

**Affiliations:** 1 Faculty of Medicine and Surgery, University of Santo Tomas, Manila, Philippines; 2 College of Rehabilitation Sciences, University of Santo Tomas, Manila, Philippines; 3 Department of Education, Graduate School, Ewha Womans University, Seoul, Republic of Korea; 4 University of Santo Tomas Hospital, Manila, Philippines

**Keywords:** COVID-19, BCG, Vaccination policy, Systematic review

## Abstract

**Background:**

COVID-19 vaccination has been advocated as the most effective way to curb the pandemic. But with its inequitable distribution and slow rollout, especially in low- to middle- income countries, it will still take a long time before herd immunity is achieved. Alternative measures must therefore be explored to bolster current COVID-19 vaccination efforts. In particular, the Bacille Calmette-Guerin vaccine has been studied extensively as to its proposed conferment of non-specific immunity against different infections, including COVID-19. The aim of this study, therefore, is to evaluate the current evidence on the effectiveness of national BCG vaccination policies in reducing infection and mortality of COVID-19.

**Methods:**

A systematic review was conducted between April to August 2021 following the Preferred Reporting Items for Systematic Reviews and Meta-analysis (PRISMA-P) guidelines. Literature was retrieved from PubMed, Cochrane, HERDIN, Web of Science, EBSCO, and Western Pacific Region Index Medicus (WPRIM). Studies conducted from January 2020 to August 2021 that fell within Level 1A to 2C of the Oxford Center for Evidence-Based Medicine were included in the review. Quality assessment was performed using the appropriate Joanna Briggs Institute critical appraisal tool and a quality assessment checklist for ecological studies adapted from Betran et al.

**Results:**

A total of 13 studies were included in this review. Nine studies reported significant association between BCG vaccination policies and COVID-19 outcomes, even when controlling for confounding variables. In addition, among other mandated vaccines, such as pneumococcal, influenza, diphtheria-tetanus-pertussis, and measles, only BCG vaccination showed significant association with decreased COVID-19 adverse outcomes. However, other factors also showed positive association with COVID-19 outcomes, particularly markers of high economic status of countries, higher median age, and greater population densities.

**Conclusion:**

The lower incidence and mortality of COVID-19 in countries with mandated BCG vaccination may not solely be attributable to BCG vaccination policies, but there is still some evidence that demonstrates a possible protective effect. Clinical trials must be continued before recommendations of BCG vaccinations are to be used as an alternative or booster vaccine against COVID-19.

**Supplementary Information:**

The online version contains supplementary material available at 10.1186/s41256-022-00275-x.

## Background

The Coronavirus disease 2019 (COVID-19) caused a global pandemic that brought the whole world to a halt. National governments instituted sweeping lockdowns, rigorous physical distancing, and aggressive contact tracing in an effort to stop the spread of the virus. Although most cases arise from asymptomatic individuals, severe COVID-19 proves very fatal because it has long term effects on the lungs, heart, and even the central nervous system [1]. As of January 2022, COVID-19 has now reached 379.09 million cases worldwide with 5.70 million deaths [2]. As with previous health crises such as polio and measles, one of the most effective ways to curb their spread is through vaccination. Vaccines have been shown to be the safest method to prevent COVID-19 by directly conferring immune protection to inoculated individuals as well as indirectly through herd immunity [3–5]. Herd immunity is defined as the indirect protection conferred by a large proportion of immune individuals to susceptible individuals against infection [4, 6, 7]. The herd immunity threshold for the initial SARS-CoV-2 virus, or the proportion of immune individuals needed to observe a decline in incidence of infection, has been estimated to be 67% of every country’s population [3, 4, 6, 7]. Due to evolving variants, the virus increased its transmissibility by more than 50%, giving rise to the need to increase the herd immunity threshold to up to 80% of the population [8].

In the hopes of permanently putting a stop to the pandemic, a multinational race began to produce vaccines capable of building herd immunity. Countries such as the United States, Russia, Germany, and China, have put forward their own vaccines which have shown varying effectiveness in preventing COVID-19 and reducing its severity and mortality. Prominent vaccines currently administered are Pfizer-BioNtech, Moderna, AstraZeneca, Gamaleya, and Sinovac with each having been granted emergency use authorization [9].

Despite this progress, there are still significant barriers that must be faced before reaching herd immunity. As of January 2022, there are 4.16 billion fully vaccinated individuals, which only comprise 52.5% of the world population even with 31.92 million vaccines administered each day [2]. These data attest that achieving herd immunity may still be far ahead. One of the most significant challenges with vaccination is vaccine inequity. New COVID-19 cases and deaths continue to rise in places with high community transmission and low vaccination coverage, which may also lead to eventual vaccine resistance [10]. Despite the high rollout of vaccines in high- and upper-middle income countries, low- and middle-income countries (LMIC) lag far behind in terms of vaccine distribution. Only 41% of people in LMICs have received at least one dose as of January 2022, compared to the 74.8% of upper-middle income countries [2, 11]. In addition to vaccine inequity, there are still issues on vaccine hesitancy and questions regarding the vaccine efficacy of current vaccines against emerging COVID-19 variants [12]. These factors may further prolong the road to herd immunity, especially in LMICs. Although COVID-19 vaccines present the most viable way to end the pandemic, other strategies have to be explored as possible adjunct measures to bolster the current vaccination effort.

One such strategy that has been heavily studied is the possible role of Bacille Calmette-Guerin vaccine, otherwise known as BCG, against COVID-19. BCG is a live attenuated vaccine derived from *Mycobacterium bovis* and is the only vaccine approved for use in the prevention of pulmonary and extra-pulmonary tuberculosis [13]. Aside from tuberculosis, studies have proposed that BCG provides non-specific immunity against non-tuberculous infections and some immunotherapeutic benefit to malignancies such as non-muscle invasive bladder cancer [13, 14].

This could potentially explain the delayed COVID-19 spread in low-income countries, where BCG vaccination is currently mandated [15]. Some properties of BCG vaccines can also support COVID-19 vaccine design, such as its established safety profile, easy accessibility, and better public acceptance [16–18]. Based on these factors, it could be used as a model for future vaccine development [13, 14, 16–18] The aim of this study, therefore, is to evaluate the current evidence on the effectiveness of national BCG vaccination policies in reducing infection and mortality of COVID-19 and discuss the continued importance of BCG vaccines.

## Methods

### Study design

This systematic review was conducted in accordance with the Preferred Reporting Items for Systematic Reviews and Meta-Analyses (PRISMA-P) as outlined by Shamseer et al. [19]. This protocol was published previously [20] and was registered with the International Prospective Register for Systematic Reviews (PROSPERO ID: CRD40221244060, Additional file 1). Ethics approval was not required due to the nature of the study.

### Study setting

This study reviewed literature that reported on BCG vaccination policies and its association with COVID-19 severity and mortality. Literature was classified according to BCG vaccination policies of countries according to the BCG World Atlas, Our World in Data, and the World Health Organization (WHO).

### Study period

Studies included in the review were those published between January 2020, which was when COVID-19 was initially reported to the WHO, until July 2021. Literature search was conducted from April to August 2021.

### Search strategy

**Fig. 1:**
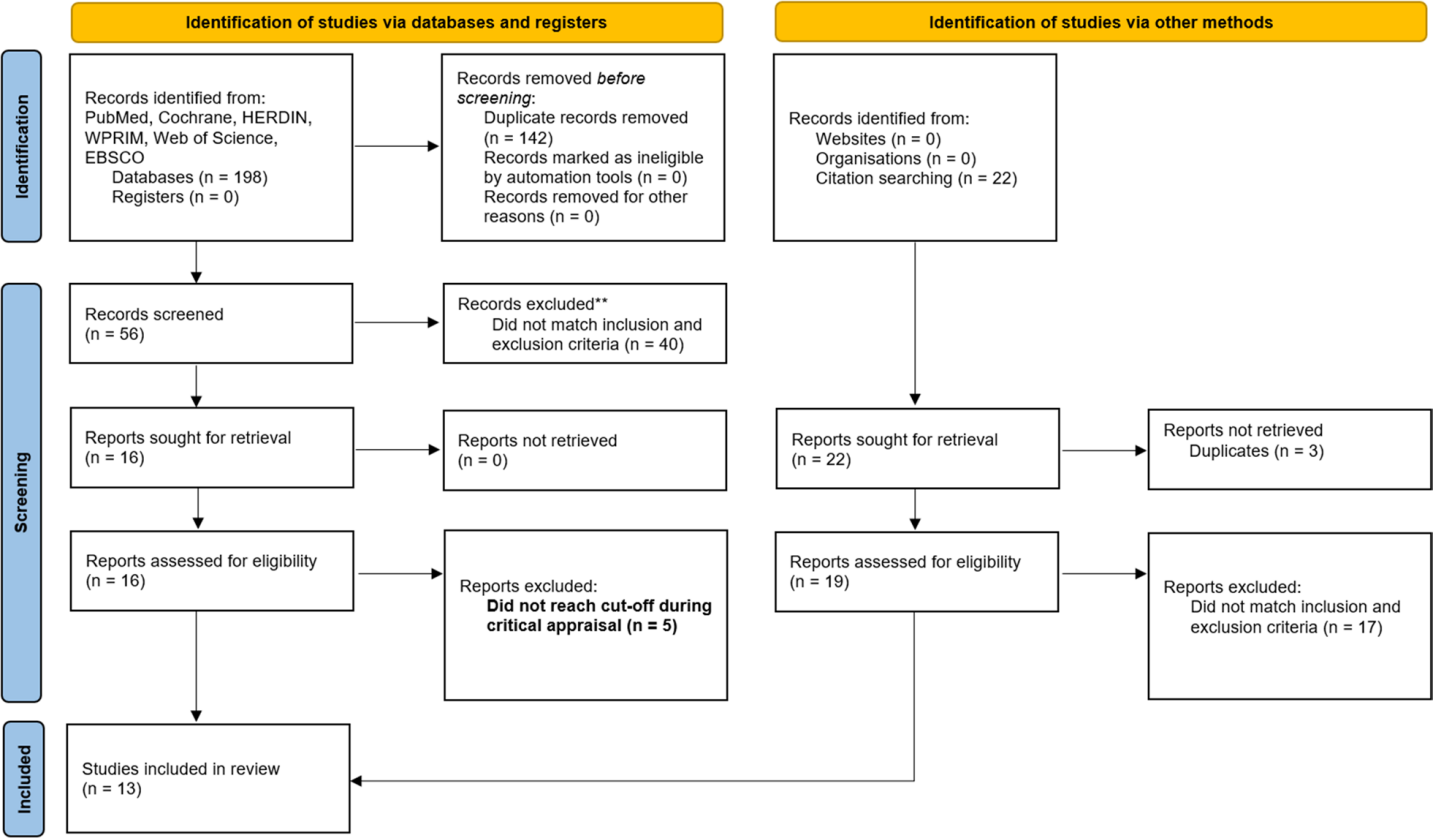
PRISMA flow diagram for searches of databases, registers, and other sources

Search terms were generated using the Population/Intervention/Comparison/Outcomes (PICO) approach, guided by the research question “What is the effectiveness of national BCG vaccination policies in reducing infection and severity of COVID-19 in their native population?” Databases included PubMed, Cochrane, HERDIN, Web of Science, EBSCO, and Western Pacific Region Index Medicus (WPRIM). The search terms used are indicated in Table 1. Snowball search was employed by manual review of citations and reference sections of the studies for additional relevant articles [21]. Figure 1 shows the study selection flow chart following the PRISMA-P guidelines.

**Table 1:** Detailed search and selection strategy

PICO component	
Population	Native population of countries with national BCG vaccination policies
Intervention	National Bacille Calmette-Guérin (BCG) policy Vaccine for tuberculosis (TB) disease
Outcome	Decrease Infection incidence and reduction of severity
Search terms	#1 Universal BCG vaccination #2 BCG policy #3 Bacille Calmette-Guérin #4 policy #5 COVID 19 #6 SARS COV 2 #7 TB Vaccine policy
Search combinations	((“Universal BCG vaccination”) OR (“BCG Policy”) AND ((“COVID 19”) OR (SARS COV 2”)) ((“Bacille Calmette-Guerin”) AND (“Policy”)) AND ((“COVID 19”) OR (SARS COV 2”)) ((“BCG Policy”) OR (“TB Vaccine Policy”)) AND ((“COVID 19”) OR (SARS COV 2”))
Databases	PubMed, Cochrane, HERDIN Plus, WPRIM, Web of Science, EBSCO

### Inclusion and exclusion criteria

Articles included studies classified under Level 1A to 2C of the Oxford Center for Evidence-Based Medicine (2009) [22]. This included longitudinal studies, randomized controlled trials, ecological studies, and systematic reviews. Descriptive studies, commentaries, editorials, ongoing research, unpublished studies, and other working papers were excluded from the study. Articles not written in English, those published prior to January 2020, or those unavailable in full text were excluded. Search results were tabulated in Google Sheets for duplicate removal. JCO, MS, CE, JT, and MJMP individually screened the studies for eligibility. Any disagreements were resolved via consensus or discussion (Additional file 2).

Studies that met the inclusion criteria were assessed by two authors regarding methodological quality using the Joanna Briggs Institute critical appraisal instruments appropriate for the study design. A quality assessment checklist adapted from Betran et al. was used to appraise ecological studies (Additional file 3) [23]. The researchers agreed on a cut-off score of 70% of the total items of the quality assessment tools to be included in the review [24]. Disagreements between two reviewers were resolved by having a third reviewer assess the study. Further disagreements were resolved through discussion and consensus (Additional file 4).

### Data extraction and synthesis

Extracted data from the selected studies were tabulated via Google sheets. Data included the authors, type of study, month and year of publication, population (countries included for ecological studies), source of BCG vaccination policy status, source of COVID-19 data, outcomes and confounding factors included, and the results of the study. JCO and CTE checked the accuracy of the recorded data.

Data synthesis consisted of comparing and contrasting the results of the outcomes and was interpreted using descriptive and inferential statistics applicable in the studies. Results of confounders were also discussed to better understand if the association between BCG vaccination policies and COVID-19 infection and severity was affected by other factors.

## Results

A total of 220 studies were identified on initial literature search. After screening for duplicates and eligibility, 38 studies were sought for retrieval. Upon quality assessment, 13 studies were included in the final review. Figure 1 shows the PRISMA flow diagram depicting the literature search. Of the 13 studies, 11 were ecological and 2 were cohorts. Table 2 shows the characteristics of the studies included in the review. Studies were further categorized into those that used statistical correlation tests (e.g., Spearman, Pearson, etc.) and those that used regression analysis.

**Table 2:** Characteristics of reviewed studies

No	References	Study design	Population	Data source		Outcome
				BCG vaccination status	COVID-19 data	
1	Escobar et al. (2020) [25]	Ecological study	USA, Europe	BCG World Atlas	Our World in Data	COVID-19 mortality
2	de Chaisemartin and de Chaisemartin (2021) [26]	Cohort	Sweden	Public Health Agency of Sweden	Public Health Agency of Sweden	COVID-19 morbidity
3	Chimoyi et al. (2020) [27]	Ecological study	107 countries	BCG World Atlas	WHO Situation Report, Our World in Data	COVID-19 mortality
4	Wickramasinghe et al. (2020) [28]	Ecological study	225 countries	BCG Atlas, WHO data on immunization coverage	Our World in Data	COVID-19 incidence and mortality
5	Szigeti et al. (2020) [29]	Ecological study	68 countries	BCG World Atlas	Our World in Data	COVID-19 mortality, incidence, case fatality
6	Li (2021) [30]	Ecological study	Worldwide	Our World in Data	Our World in Data	COVID-19 morbidity and mortality
7	Berg et al. (2020) [31]	Ecological study	135 countries	BCG World Atlas	Our World in Data	COVID-19 incidence and mortality
8	Ogimi et al. (2021) [32]	Ecological study	140 countries	BCG World Atlas	Our World in Data	COVID-19 mortality
9	Ebina-Shibuya et al. (2020) [33]	Ecological study	171 countries	BCG World Atlas	Worldometer	COVID-19 mortality
10	Brooks et al. (2021) [34]	Ecological study	57 countries	BCG World Atlas	Our World in Data	COVID-19 mortality
11	Abdulah and Hassan (2021) [35]	Ecological study	186 countries	WHO website	WHO Website	COVID-19 incidence and mortality
12	Rivas et al. (2021) [36]	Cohort	USA	Cedars-Sinai Medical Network	Cedars-Sinai Department of Pathology	Seroprevalence of anti-SARS-COV2 IgG, incidence of self-reported symptoms
13	Klinger et al. (2020) [37]	Ecological study	Worldwide	BCG World Atlas	Worldometer	COVID-19 deaths per million, cases per million, hospitalization with serious and critical conditions, recovered

### Correlation between BCG vaccination and COVID-19 morbidity and mortality

Studies that used statistical correlation methods were synthesized together to assess the correlation between BCG policies and COVID-19 outcomes (Table 3). Four studies were included in this assessment. Statistical tests used by reviewed studies were either Spearman rho [28, 29] or Pearson [30, 37]. Two of the studies showed no significant correlation between mandatory BCG vaccination and COVID-19 outcomes while two studies showed strong correlation between BCG vaccination and decreased COVID-19 outcomes.

**Table 3:** Effects of BCG Vaccination on morbidity and mortality based on correlation

References	Statistical analysis	Variables	Results
Wickramasinghe et al. (2020) [28]	Spearman correlation	BCG coverage COVID-19 cases and deaths per 100,000	No significant correlation between BCG immunity and COVID-19 cases or deaths per 100,000
Szigeti et al. (2020) [29]	Spearman correlation	BCG vaccination Daily rates of COVID-19 case fatality	No significant correlation between year of BCG policy establishment and mortality rates
Li (2021) [30]	Pearson's correlation	BCG vaccination rates Diphtheria tetanus toxoid and pertussis immunization (DTP) COVID-19 death	Highly significant inverse correlation between BCG vaccination rate and COVID-19 deaths; no significant correlation between DTP vaccination and COVID-19 deaths
Klinger et al. (2020) [37]	Pearson's correlation	BCG administration years COVID-19 death and cases per million	Strong negative correlation between deaths and cases per million and years of BCG administration

### Association between BCG vaccination and COVID-19 morbidity and mortality

**Fig. 2:**
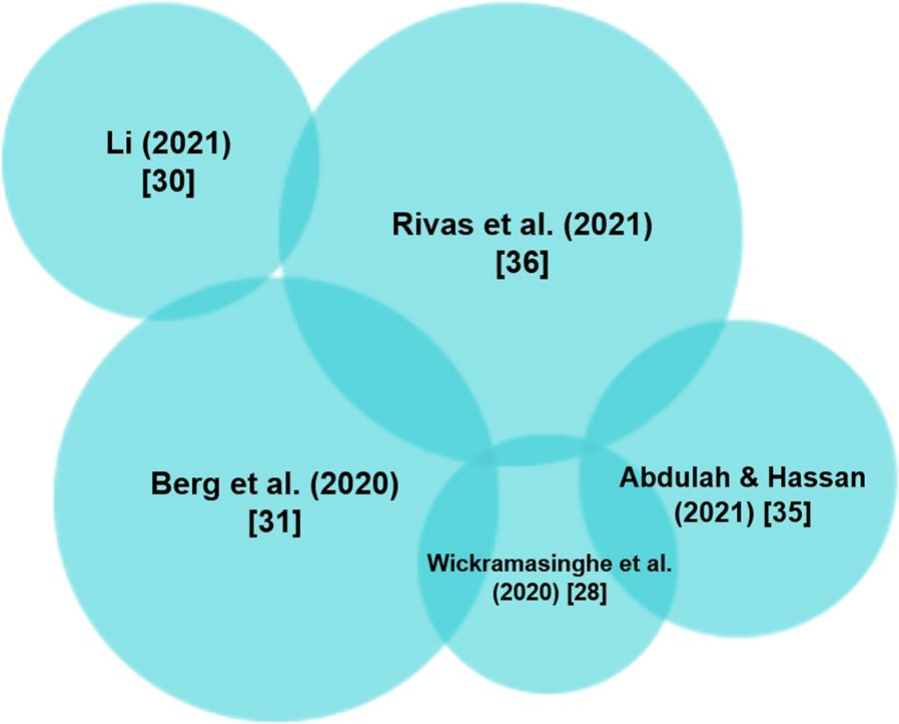
Heat map of the magnitude of association between presence of national BCG Vaccination Policies and COVID-19 morbidity

**Fig. 3:**
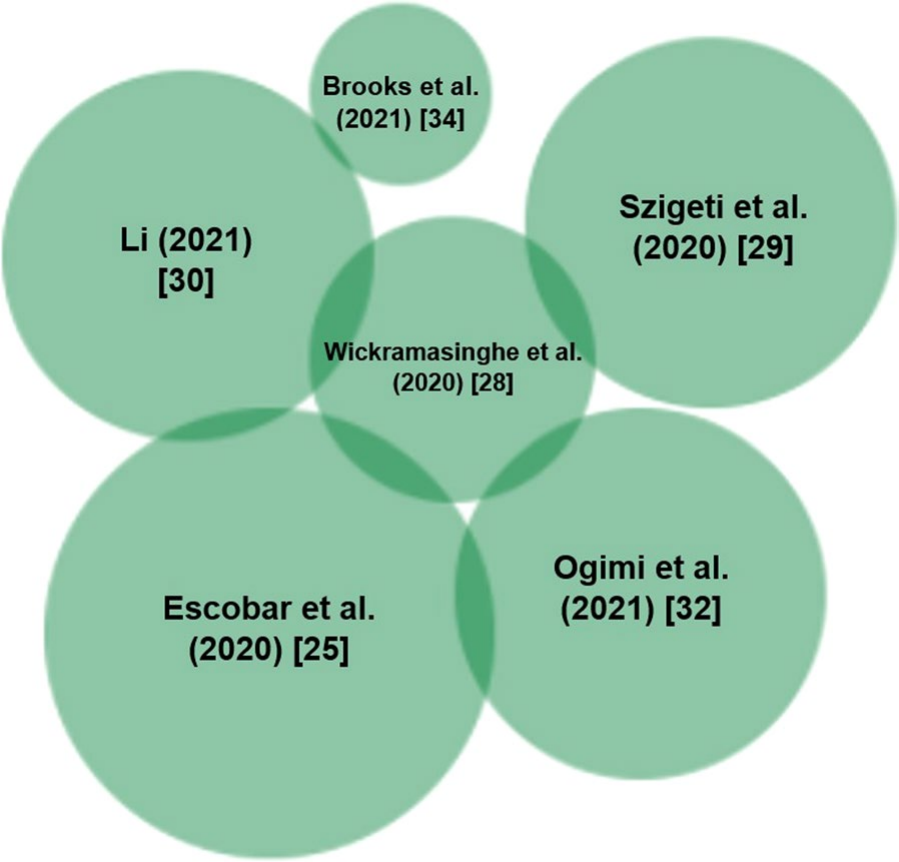
Heat map of the magnitude of association between presence of national BCG Vaccination Policies and COVID-19 mortality

To assess for overall cause-and-effect, studies that used regression analysis were grouped together and compared. Table 4 presents the findings of the studies included. Of the 12 studies that used regression analysis, only 3 studies did not find a significant association between BCG vaccination and COVID-19 outcomes [26, 27, 35]. One study found a significant association between BCG vaccination and COVID-19 deaths per million but found no significant association between BCG vaccination and COVID-19 deaths per case [29]. Figures 2 and 3 show the magnitude of association between BCG vaccination policies and COVID-19 morbidity and mortality. These studies indicate the large magnitude of association between vaccination policies and COVID-19 outcomes, which may demonstrate the protective effect of such policies.

**Table 4:** Effects of BCG vaccination on morbidity and mortality based on association and confounding variables

References	Statistical analysis	Confounding variables	Results
Escobar et al. (2020) [25]	Multiple linear regression	Stage of COVID-19 pandemic Development Rurality Population density Age structure	Inverse association between BCG vaccination and COVID-19 mortality after controlling for confounding variables
de Chaisemartin and de Chaisemartin (2021) [26]	Regression discontinuity	None reported due to cohort design, no variable cross-country comparisons	Universal BCG policy has no effect on COVID-19 cases, hospitalizations, and deaths per 1000 inhabitants
Chimoyi et al. (2020) [27]	Log-linear regression	GDP per capita Population size Population > 65 years Tests per capita Stringency level at 100th case Smoking prevalence Difference between date of 100th case and end of May 2020	No significant association between BCG status and cases and deaths even when adjusted for different time points Positive association seen in population size, population > 65 years, and tests per capita
Wickramasinghe et al. (2020) [28]	Linear regression	Income level	Income status had a statistically significant effect on caseload; universal BCG vaccination policy had a significant inverse effect
Szigeti et al. (2020) [29]	Multiple regression	Historic colonization status Median age Urban population percentage Population density Air passengers	Deaths per million as dependent variable: BCG vaccination was statistically significant Deaths per case as dependent variable: BCG vaccination was non-significant ‘Historic colonization status’, median age were statistically significant
Berg et al. (2020) [31]	Linear mixed effect model	Cultural dimensions Individualism versus collectivism Power distance	Countries with BCG vaccination policy until at least 2000 had significantly slower growth rate of COVID-19 Significantly lower growth rate of COVID-19 deaths in countries with BCG vaccinations until at least 2000
Ogimi et al. (2021) [32]	Nested linear model	Measles vaccine coverage (MCV) Healthcare Access and Quality Index (HAQI) Life expectancy Number of hospital beds Physicians per population GDP	BCG was marginally associated with lower COVID-19 death rates. Increased association between BCG and COVID-19 as a function of HAQI MCV was also associated with lower COVID-19 deaths, but no significant association when adjusted for HAQI
Ebina-Shibuya et al. (2020) [33]	Multiple regression	Gross national income, Life expectancy at birth Infant mortality at 1st year Population density Annual average temperature	Controlling for confounders, countries without BCG policy have increased COVID-19 associated mortality compared to those with BCG policy
Brooks et al. (2021) [34]	Linear regression	GDP Population density Total population Population over 65 Average temperature Stringency index Polio and measles vaccine coverage	BCG vaccination was consistently associated with lower COVID-19 related mortality
Abdulah and Hassan (2021) [35]	Generalized linear model	Health system indicators Social insurance Social assistance Mandatory individual account system Economic status based on the World Bank MCV, DTP3, PCV vaccination	No significant association between infection and mortality and BCG vaccination
Rivas et al. (2021) [36]	Logistic regression	Pre-existing comorbidities Age and sex Pneumococcal, meningococcal, influenza vaccination	Decreased incidence of medically diagnosed or RT-PCR positive COVID-19 in those with history of BCG vaccination Controlling for age, sex and comorbidities, less seroconversion and significantly lower anti-SARS-CoV-2 IgG index were seen in those with history of BCG vaccination
Klinger et al. (2020) [37]	Linear regression	Quarantine status Economic development indicators Chronic disease prevalence Death rate from cardiovascular disorders	BCG administration years is consistently within the top two most significant coefficients compared to 23 confounding variables and ranks high in terms of coefficient effect

### Effects of confounding variables

Even after controlling for confounding variables, most studies still found a significant association between BCG vaccination policies and decreased COVID-19 outcomes. The list of confounding variables identified per study is presented in Table 3. Positive associations were consistently observed between demographic variables, such as higher median age [29–31, 37] proportion of population > 65 years [25, 27, 28, 34], population density [25, 30, 31, 34], and COVID-19 outcomes. Evidence of a country’s higher economic status, namely high gross domestic product (GDP), high gross national income (GNI), and high World Bank classification, was consistently associated with higher COVID-19 incidence and mortality [28–30, 34, 35]. ‘Historic colonization status,’ an arbitrary variable classifying countries as to their history of participation in colonization efforts in the late 1400 s, was also used as a marker for advanced economies and was strongly associated with COVID-19 outcomes [29].

Some studies also observed relationships between other mandated vaccination programs and COVID-19 outcomes. Diphtheria, tetanus, and pertussis (DTP) coverage was not seen to be significant in decreasing COVID-19 outcomes [30, 35]. In two studies, measles vaccination coverage either showed a significant albeit weaker association to decreased COVID-19 outcomes compared to BCG or no association at all [32, 34, 35]. Pneumococcal, meningococcal, and influenza vaccinations were also assessed and were shown to have no significant effect on COVID-19 outcomes [36]. Lastly, oral polio vaccination showed no significant association to COVID-19 outcomes [34].

## Discussion

### Summary of the findings

Overall, most studies indicate an inverse association between BCG vaccination policies and COVID-19 incidence and mortality. However, this review presents a different finding from the study done by Ricco et al. early on in the pandemic, which previously concluded that there is no sound evidence to recommend BCG vaccination for the prevention of COVID-19 [38]. Findings may result from the inclusion of more recent studies on the topic, including those from early to mid-2021 [30, 32, 34, 35]. For one, Ricco et al. report that only two out of the 14 studies reviewed controlled for confounding variables. Meanwhile, most studies included in this review controlled for some or all confounding variables reported. In addition, Ricco et al. point out that COVID-19 outcomes highly depend on the reliability of reporting COVID-19 cases and deaths. Although consistent with other studies [29, 39], this may have been a more significant factor in the early phases of the pandemic because of its more erratic and uncertain course at the time [40]. As the pandemic continued, more robust testing and reporting strategies were implemented [30, 41], thus increasing the raw data quality.

### Nonspecific protective effect of BCG vaccination

Numerous studies have proposed the nonspecific immune effect of BCG on unrelated infections as a possible mechanism to explain its protective effect against COVID-19. BCG has long been reported to grant "trained immunity," a nonspecific innate immune response resulting from epigenetic reprogramming. Through epigenetic reprogramming, BCG modifies human monocytes leading to increased cytokine production. This epigenetic programming explains BCG's ability to confer protection against unrelated infections [42, 43]. In addition, possible long-term effects are brought about by heterologous T helper 1 (Th1) and Th17 immune responses [43] and more rapid seroconversion from enhanced leukocyte response and robust cytokine response to unrelated pathogens. This long-term effect may explain the protection BCG confers on individuals years after vaccination, which is significant since policies usually mandate its inoculation immediately after birth.

Studies included in this review have demonstrated this nonspecific protective effect. Rivas et al. explored the serologic effects of previous BCG vaccination on COVID-19 [36]. The study demonstrated that a history of BCG vaccination in healthcare workers lowers the incidence of clinically or laboratory-confirmed diagnosis of COVID-19. The study also demonstrated that prior BCG vaccination was associated with lower seroconversion and anti-SARS-CoV-2 IgG index even when controlled for age and sex [36]. These lower serological features are still consistent even in the presence of co-morbidities, even though these conditions increase the likelihood of acquiring COVID-19. This study, therefore, gives credence to the supposed nonspecific protection afforded by prior inoculation with BCG.

Compared to other vaccines, only BCG has consistently shown a robust significant association in decreasing COVID-19 outcomes. Studies reported on DTP, meningococcal, pneumococcal, measles, influenza, and oral polio against COVID-19 outcomes have failed to produce the same protective effect compared to BCG [30, 32, 34–36]. Results may further illustrate that BCG vaccination alone could confer nonspecific immunity, thereby reducing incidence and mortality related to COVID-19. This stresses the importance of BCG vaccines, not only for its potential against COVID-19, but also against other infectious diseases and non-communicable diseases such as malignancies [13]. Additional studies on BCG must be done to augment these findings, not only to help eradicate tuberculosis but also to increase its potential as a model for future vaccine design [13, 17, 18].

### Confounding variables and lower COVID-19 adverse outcomes

This study is the first to synthesize the effects of the common confounding variables reported among studies on BCG and COVID-19 adverse outcomes. Although most of the studies controlled for these variables, their effect on a higher incidence of COVID-19 may still have played a role in the perceived effectiveness of BCG policies against COVID-19.

Markers of the high economic status of a country (e.g., GDP, GNI, World Bank classification) have consistently been associated with increased COVID-19 incidence and mortality [28–30, 34, 35]. Those higher-income countries are also likely to have phased out their mandated BCG policies [27, 28, 35]. However, it is essential to note that these higher-income countries are likely to be more developed and may have better healthcare infrastructure, thus enabling adequate testing and contact tracing measures [29, 30, 32]. Consequently, this increases the recorded COVID-19 adverse outcomes in these countries compared to those of lower-income countries, which may suffer from underreporting of cases. This may have contributed to the relatively decreased incidence in countries with a current mandated BCG policy [29, 30] and may therefore underscore the protective effect of BCG against COVID-19 outcomes.

Studies have also reported positive associations between specific population demographics and COVID-19 adverse outcomes. For example, countries with higher median age and population over 65 have shown an increase in COVID-19 incidence and mortality [25, 27–31, 34, 36]. These two factors show that a higher proportion of older residents in these countries may have increased COVID-19 adverse outcomes resulting in more susceptibility to acquiring COVID-19 and exhibiting severe manifestations leading to death [44]. Furthermore, in relation to this study, an older-aged population also reflects these countries' higher economic status, which may have also contributed to the increased incidence and mortality to COVID-19 in these high-income countries [28, 30].

### Significance of the findings

This review was able to collate results from various papers about the effectiveness of BCG vaccine policies with COVID-19. Unlike the previous review by Ricco et al., the current study was able to find evidence that supports the protective effect of such policies against COVID-19. The study was also able to account for the influence of various confounding variables in the association. However, due to most studies being ecological or observational, actual cause-and-effect between BCG and COVID-19 incidence and mortality cannot be ultimately concluded. The studies reviewed also point out the possibility that the supposed protective effect may have merely been due to the effect of other variables.

This study, therefore, highlights the importance of conducting clinical trials to determine the effectiveness of an intervention adequately. Short papers and case studies on the topic have shown conflicting results, demonstrating either no protective effect [45, 46] or some effect such as lower hospitalization rates [47]. Studies on animal models have also been performed which may provide physiologic evidence for the perceived protective effect [48]. Recently completed randomized controlled trials are now attesting to the protective effect of BCG, supporting the findings of this study [49–51]. In contrast, some early clinical trials show no effect of BCG vaccination on COVID-19 infection [52, 53]. Because of the conflicting nature of the results, these data should be interpreted with utmost prudence [54]. It is therefore still recommended that further studies be continued to map the true effect of BCG vaccination against COVID-19, especially with the rise of variants and the possibility of waning immunity with the current vaccines [55]. Studies have now even shifted to explore the possibility of using BCG as a booster for current COVID-19 vaccines [56]. Further studies to be performed at the molecular or cellular level may also provide further basis for this protective effect. These can help in conclusively establishing a link between BCG vaccination and lower COVID-19 adverse outcomes and may also aid in the production of future vaccines.

### Limitations and recommendations

This study has several limitations. For one, our database was only limited to open access journals and those that were published in English. Moreover, our study data gathering was only limited until August 2021. With the COVID-19 situation continuously evolving and with even more papers being published regarding this disease, recent breakthroughs published after the said date might not have been included in this study. We recommend future researchers to include recent data and information not covered in this study.

## Conclusion

Even though the lower incidence and mortality due to COVID-19 may not merely be attributable to BCG vaccination policies, there is still a large amount of evidence that shows their possible protective effect. Only actual clinical trials, which are just recently being completed, may truly be able to elucidate the actual effect of BCG vaccination against COVID-19. Therefore, before recommendations can be made regarding the use of BCG vaccination as an adjunct solution to the pandemic, further studies must be done to adequately report its efficacy, including further review of these clinical trials. Lastly, further research to improve BCG vaccines must be continued to strengthen its effectiveness in not only eliminating tuberculosis but also in aid of reducing the morbidity and mortality of other communicable and non-communicable diseases.

## Supplementary Information

**Additional file 1**. PROSPERO Registration.

**Additional file 2**. Initial record screening.

**Additional file 3**. Quality assessment checklist for ecological studies adapted from Betran et al.

**Additional file 4**. Critical appraisal of studies using the adapted appraisal tool from Betran et al. and the JBI Critical Appraisal Instruments.

## Data Availability

All data generated or analyzed during this study are included in this published article and the Additional files 1–4.

## Abbreviations

BCG: Bacille Calmette-Guerin vaccine
COVID-19: Coronavirus disease 2019
DTP: Diphtheria-tetanus-pertussis vaccine
GDP: Gross domestic product
GNI: Gross national income
HAQI: Healthcare Access and Quality Index
IgG: Immunoglobulin G
LMIC: Low- and middle-income country
MCV: Measles-containing vaccine
PCV: Pneumococcal conjugate vaccine
PRISMA-P: Preferred Reporting Items for Systematic Reviews and Meta-Analysis Protocol
Th1: T helper 1 cell
Th17: T helper 17 cell
SARS-CoV-2: Severe acute respiratory syndrome coronavirus 2
WHO: World Health Organization
WPRIM: Western Pacific Region Index Medicus
