# Supplementary material for: Effectiveness of Bacille Calmette-Guerin vaccination policies in reducing infection and mortality of COVID-19: a systematic review
Source: Glob Health Res Policy. 2022 Nov 7;7:42. doi: 10.1186/s41256-022-00275-x (PMC9638327; doi:10.1186/s41256-022-00275-x)
Supplement: Supplementary file 1 — Additional file 1. PROSPERO Registration. [file 41256_2022_275_MOESM1_ESM.pdf]

Citation

Joseph Christian Obnial, Mystie Suzuki, Janine Trixia Austria, Ma. Jamaica Monique Ponce, Catherine Joy Escuadra, Elaine Cunanan. Systematic review on the effectiveness of BCG vaccination policies in reducing infection, severity and mortality of COVID-19. PROSPERO 2021 CRD42021244060 Available from: https://www.crd.york.ac.uk/prospero/display_record.php?ID=CRD42021244060

Review question [1 change]

What is the effectiveness of national BCG vaccination policies in reducing infection, severity, and mortality of COVID-19 in their native population?

Searches

The following databases will be used to identify the studies to review

- PubMed

- Cochrane Central Register of Controlled Trials (CENTRAL)

- Herdin Plus

- Western Pacific Region Index Medicus

- Web of Science

- EBSCO

Search dates: April 1 to April 30, 2021

Restrictions: Language (English), Year (January 2020-2021)

Search Re-run: Yes

Unpublished papers: No

Types of study to be included

Level 1A to 2C for therapy/prevention questions using the Oxford Center for Evidence-Based Medicine Levels of Evidence table (https://www.cebm.ox.ac.uk/resources/levels-of-evidence/oxford-centre-for-evidence-based-medicine-levels-of-evidence-march-2009).

Descriptive studies, commentaries, editorials, ongoing research, unpublished studies, and other working papers are excluded from the study.

Condition or domain being studied

COVID-19 - is the disease caused by a new coronavirus called SARS-CoV-2. WHO first learned of this new virus on 31 December 2019, following a report of a cluster of cases of ‘viral pneumonia’ in Wuhan, People’s Republic of China

Participants/population

Native population of countries with national BCG vaccination policies

Intervention(s), exposure(s)
Bacille Calmette-Guérin (BCG) vaccine - a vaccine for tuberculosis (TB) disease

Comparator(s)/control
No BCG vaccine

Context [1 change]

Studies that involved assessment of infection and severity (including mortality rate) of COVID-19 in countries with national BCG vaccination policies

Main outcome(s) [1 change]

Decrease in infection incidence and reduction of severity and mortality of COVID-19

Measures of effect

Relative risks, odds ratio, risk difference, or other measure of effects computed in the study.

Additional outcome(s)
None

Data extraction (selection and coding)
Six researchers will individually screen the studies obtained during the formal literature search against the eligibility criteria. Disagreements will be resolved through consensus or discussion.

Studies that passed through the initial screening will undergo another round of screening using critical appraisal. Two reviewers will independently appraise each article using the appropriate tool for the study design. Reviewers will be blinded to avoid any potential bias. Any disagreements will be resolved by a third reviewer. The final list of articles will be recorded via Google sheets.

Six researchers will then extract data from the selected studies including: population details (including demographics and baseline characteristics), intervention details, study design and methodology, and outcomes relevant to the research question. Two researchers will check the accuracy of the recorded data.

Missing Data will be sought out by contacting the original investigators of the study via email. All data extracted will be recorded through Google and Excel spreadsheets.

Risk of bias (quality) assessment [1 change]

Studies meeting the eligibility criteria will be assessed by two authors as to their methodological quality using the Joanna Briggs Institute critical appraisal instruments appropriate for the study design. For ecological studies, the quality assessment tool by Betran et al. (2015) was adapted for use in this study. The researchers pre-established the minimum standard or passing score of 70% of the total items of the checklists to be included in the review. Studies below 70% on the appropriate appraisal tool will be deemed of low methodological quality and would therefore be excluded. During instances when scores resulted in disagreement between the two reviewers, a third researcher will be tasked to assess the study. Any further disagreements between their ratings will be resolved through discussion and consensus.

Reference:

Aromataris E, Munn Z (Editors). JBI Manual for Evidence Synthesis. JBI, 2020. Available from https://synthesismanual.jbi.global. https://doi.org/10.46658/JBIMES-20-01

Betran, A.P., Torloni, M.R., Zhang, J. et al. What is the optimal rate of caesarean section at population level? A systematic review of ecologic studies. Reprod Health 12, 57 (2015). https://doi.org/10.1186/s12978-015-0043-6

Strategy for data synthesis [2 changes]

All of the following relevant data will be extracted and synthesized from the studies that passed the critical appraisal phase:

- Study design

- Setting

- Participants

- Sampling strategy

- Assessment tools

- Description of BCG vaccine policy

- Data Gathering Procedures

- Analyses

- Results (infection incidence, severity of infection, mortality rates)

Researchers will then further compare and contrast descriptive and inferential statistics that will be reported by the studies, including the measures of effects (eg. effect size, odds ratio, risk ratio, confidence intervals etc). Any discrepancies will be discussed and clarified by all authors. Quantitative synthesis (using tables and if applicable graphs) will be done considering the sample, BCG vaccine policy classification according to BCG atlas 2020 (Source: http://www.bcgatlas.org/index.php?fbclid=IwAR0spBn4xDY_j8QmKC59AnVjvyI01QRiaJvKv3HvV7N846el_3htGRZ-Xg4), methodology, and study quality. No minimum number of studies will be set for synthesis as authors aim to utilize all currently available published studies.

Analysis of subgroups or subsets

None

Contact details for further information

Joseph Christian Obnial
josephccobnial@gmail.com

Organisational affiliation of the review

University of Santo Tomas, Manila, Philippines
https://www.ust.edu.ph/

Review team members and their organisational affiliations [2 changes]

Mr Joseph Christian Obnial. University of Santo Tomas
Ms Mystie Suzuki. University of Santo Tomas
Ms Janine Trixia Austria. University of Santo Tomas
Ms Ma. Jamaica Monique Ponce. University of Santo Tomas
Ms Catherine Joy Escuadra. University of Santo Tomas
Dr Elaine Cunanan. University of Santo Tomas

Type and method of review

Epidemiologic, Intervention, Systematic review

Anticipated or actual start date

09 April 2021

Anticipated completion date [1 change]

30 September 2021

Funding sources/sponsors

None

Conflicts of interest

Language

English

Country

Philippines

Stage of review [1 change]

Review Completed not published

Subject index terms status

Subject indexing assigned by CRD

Subject index terms

BCG Vaccine; COVID-19; Humans; Policy; SARS-CoV-2; Tuberculosis; Vaccination

Date of registration in PROSPERO

08 April 2021

Date of first submission

07 April 2021

Stage of review at time of this submission [3 changes]

**Stage**	**Started**	**Completed**
Preliminary searches	Yes	Yes
Piloting of the study selection process	Yes	Yes
Formal screening of search results against eligibility criteria	Yes	Yes
Data extraction	Yes	Yes
Risk of bias (quality) assessment	Yes	Yes
Data analysis	Yes	Yes

Revision note

- Included mortality in the title, research question, and outcomes of interest (RATIONALE - several studies reported on this and would be included in the data analysis)- Extended the review to October 2021 (RATIONALE - Review is still ongoing) - Data analysis has been started

The record owner confirms that the information they have supplied for this submission is accurate and complete and they understand that deliberate provision of inaccurate information or omission of data may be construed as scientific misconduct.

The record owner confirms that they will update the status of the review when it is completed and will add publication details in due course.

Versions

08 April 2021
14 July 2021
10 September 2021
04 November 2021
