# Supplementary material for: Effectiveness of Bacille Calmette-Guerin vaccination policies in reducing infection and mortality of COVID-19: a systematic review
Source: Glob Health Res Policy. 2022 Nov 7;7:42. doi: 10.1186/s41256-022-00275-x (PMC9638327; doi:10.1186/s41256-022-00275-x)
Supplement: Supplementary file 2 — Additional file 2. Initial record screening. [file 41256_2022_275_MOESM2_ESM.pdf]

Supplemental File 2 Initial record screening
Obnial et al.
PROSPERO ID: CRD40221244060

NO.	RECORD IDENTIFICATION METHOD	Author	DECISION	REASON
1	DATABASE	Escobar et al.	INCLUDE	
2	DATABASE	Roti et al.	EXCLUDE	Literature Review
3	DATABASE	Morrison	EXCLUDE	Commentary
4	DATABASE	Sharma et al.	EXCLUDE	Literature Review
5	DATABASE	Meena et al.	EXCLUDE	Letter to the Editor
6	DATABASE	Mohaptra and Misra	EXCLUDE	Letter to the Editor
7	DATABASE	Khade et al.	EXCLUDE	Literature Review
8	DATABASE	Sharma et al.	INCLUDE	
9	DATABASE	Hauer et al.	EXCLUDE	Letter to the Editor
10	DATABASE	Hensel et al.	INCLUDE	
11	DATABASE	Charoenlap et al.	EXCLUDE	Literature Review
12	DATABASE	Pereira et al.	EXCLUDE	Letter to the Editor
13	DATABASE	Jirjees et al.	EXCLUDE	Literature Review
14	DATABASE	Yitbarek et al.	EXCLUDE	Data overlap
15	DATABASE	Ricco et al.	EXCLUDE	Data overlap
16	DATABASE	Chaisemartin & Chaisemartin	INCLUDE	
17	DATABASE	Aksu et al.	EXCLUDE	OUT OF SCOPE
18	DATABASE	Chimoyi et al.	INCLUDE	
19	DATABASE	Wickramasinghe et al.	INCLUDE	
20	DATABASE	Kumar et al.	EXCLUDE	OUT OF SCOPE
21	DATABASE	Hidvégi & Nichelatti	INCLUDE	
22	DATABASE	Reka et al.	EXCLUDE	Literature Review
23	DATABASE	Szigeti et al.	INCLUDE	
24	DATABASE	Sayed et al.	INCLUDE	
25	DATABASE	Vashishtha	EXCLUDE	Commentary
26	DATABASE	Li	INCLUDE	
27	DATABASE	Huang et al.	EXCLUDE	No full text
28	DATABASE	De Wals et al.	EXCLUDE	Commentary
29	DATABASE	Berg et al.	INCLUDE	
30	DATABASE	Salvador et al.	EXCLUDE	OUT OF SCOPE
31	DATABASE	Samaddar et al.	EXCLUDE	Commentary
32	DATABASE	Ebina-Shibuya et al.	EXCLUDE	Letter to the Editor
33	DATABASE	Irfani et al.	EXCLUDE	OUT OF SCOPE
34	DATABASE	Cherif et al.	EXCLUDE	Commentary
35	DATABASE	Ayoub et al.	EXCLUDE	Case-study
36	DATABASE	Alyasin et al.	EXCLUDE	Commentary
37	DATABASE	Ogimi et al.	INCLUDE	
38	DATABASE	Ebina-Shibuya et al.	INCLUDE	
39	DATABASE	Brooks et al.	INCLUDE	
40	DATABASE	Marín-Hernández	EXCLUDE	Letter
41	DATABASE	Pandita et al.	EXCLUDE	Review Article
42	DATABASE	Abdulah & Hassan	INCLUDE	

Supplemental File 2 Initial record screening
Obnial et al.
PROSPERO ID: CRD40221244060

43	DATABASE	Kumarab et al.	EXCLUDE	OUT OF SCOPE
44	DATABASE	Kook	EXCLUDE	OUT OF SCOPE
45	DATABASE	Vakzine Projekt Management GmbH	EXCLUDE	OUT OF SCOPE
46	DATABASE	Madsen et al.	EXCLUDE	RCT
47	DATABASE	Universidad de Antioquia	EXCLUDE	Study not completed
48	DATABASE	Rivas et al.	INCLUDE	
49	DATABASE	Junqueira-Kipnis et al.	EXCLUDE	Study protocol
50	DATABASE	Hellenic Institute for the Study of Sepsis	EXCLUDE	RCT
51	DATABASE	Bandim Health Project	EXCLUDE	RCT
52	DATABASE	Bandim Health Project	EXCLUDE	RCT
53	DATABASE	Aaby et al.	EXCLUDE	Commentary
54	DATABASE	COVID-19 AND BCG VACCINE: IS THERE A LINK?	EXCLUDE	No full text
55	DATABASE	Banik et al	EXCLUDE	Review
56	DATABASE	Gupta	EXCLUDE	Letter to the Editor
57	DATABASE	Gupta	EXCLUDE	OUT OF SCOPE
58	DATABASE	Ayanoğlu et al.	EXCLUDE	No full text
59	DATABASE	Coronavirus impact on research spreads.	EXCLUDE	OUT OF SCOPE
60	CITATION SEARCHING	Miller et al	EXCLUDE	Pre-print
61	CITATION SEARCHING	Leentjens et al.	EXCLUDE	OUT OF SCOPE
62	CITATION SEARCHING	De Castro et al.	EXCLUDE	OUT OF SCOPE
63	CITATION SEARCHING	Hollm-Delgado et al.	EXCLUDE	OUT OF SCOPE
64	CITATION SEARCHING	Stensballe et al.	EXCLUDE	OUT OF SCOPE
65	CITATION SEARCHING	Wardhana et al.	EXCLUDE	OUT OF SCOPE
66	CITATION SEARCHING	Kleinnijenhuis et al.	EXCLUDE	OUT OF SCOPE
67	CITATION SEARCHING	Roth et al.	EXCLUDE	OUT OF SCOPE
68	CITATION SEARCHING	Akiyama & Ishida	EXCLUDE	Pre-print
69	CITATION SEARCHING	Green et al.	EXCLUDE	Pre-print
70	CITATION SEARCHING	Dolgikh	INCLUDE	
71	CITATION SEARCHING	Dayal & Gupta	EXCLUDE	Pre-print
72	CITATION SEARCHING	Hegarty et al.	EXCLUDE	Commentary
73	CITATION SEARCHING	Shet et al.	EXCLUDE	Pre-print
74	CITATION SEARCHING	Sala et al.	EXCLUDE	Pre-print
75	CITATION SEARCHING	Klinger et al.	INCLUDE	
76	CITATION SEARCHING	Singh	EXCLUDE	Pre-print
77	CITATION SEARCHING	Goswami et al.	EXCLUDE	Pre-print
78	CITATION SEARCHING	Shivendu et al.	EXCLUDE	Pre-print
