# Supplementary material for: Effectiveness of Bacille Calmette-Guerin vaccination policies in reducing infection and mortality of COVID-19: a systematic review
Source: Glob Health Res Policy. 2022 Nov 7;7:42. doi: 10.1186/s41256-022-00275-x (PMC9638327; doi:10.1186/s41256-022-00275-x)
Supplement: Supplementary file 3 — Additional file 3. Quality assessment checklist for ecological studies adapted from Betran et al. [file 41256_2022_275_MOESM3_ESM.pdf]

Obnial et al.
PROSPERO ID: CRD40221244060

Supplemental File 3: Quality assessment checklist for ecological studies adapted from Betran et al. (2015)

Evaluation criterion	Categories	Definition	Points (max=21)
**STUDY DESIGN (max=12)**			
Design	Cross-sectional<br>Longitudinal	If it is a multi-level design (e.g. ecologic + individual), the study is upgraded one point (e.g. cross-sectional + multi-level receives 2 points)	1<br>2
Sample size	< 80% units<br>≥ 80% units	Number of ecologic units included in the analysis as a proportion of the total number of units, e.g. 119 countries of a total of 180 worldwide would be 66%.	0<br>1
Unbiased inclusion of units	No<br>Yes	Were the units included representative of the group for which inferences are being drawn? For example, for worldwide inferences, inclusion of only developed countries would be biased.	0<br>1
Level of data aggregation	Other than below (City, Municipal)<br>Regional, State<br>National	Population to which the units refer to. “Other” may be: city, race groups.	1<br>2<br>3
Level of inference	Individual or unclear<br>Ecologic (Group)	Use of the results of the analysis of the study’s sample data to draw inferences for individuals or groups (ecologic).	0<br>1
Prespecification of ecologic units	No<br>Yes	Were the ecologic units selected to suit the hypothesis/objective/s? (as opposed to selection motivated by convenience or necessity)	0<br>1
Outcomes of interest included	Some<br>All	Inclusion of all relevant outcomes (i.e. infection incidence and infection severity) or only of some outcomes.	1<br>2
Source of data	Inadequate<br>Adequate	Validity of the sources of data to represent the level that it refers to (e.g. the incidence rate in one city would be an inadequate source of data to represent the national incidence rate).	0<br>1
**STATISTICAL METHODOLOGY (max=6)**			
Analytic methodology	Spearman’s rank correlation, Linear least square regression models, Quadratic model, Exponential model, LOWESS, Fractional polynomial regression Piecewise regression,	All statistical methods are acceptable as long as they are used appropriately. We assign a score based on the sophistication and flexibility of the method.<br>1 = Spearman’s rank correlation, Linear least square regression models, Quadratic model, Exponential model	1

		2 = LOWESS, Fractional polynomial regression, Piecewise regression,	2
Validity of regression	No<br>Yes	Did the adjustment have at least 10 units per covariate?	0<br>1
Use of covariates	None<br>Socio-economic<br>Socio-economic + clinical	Authors adjusted the analysis for desirable variables or not. Example of socio-economic covariates: GDP or HDI. Example of clinical covariates: proportion of other related conditions	0<br>1<br>2
Proper adjustment for covariates (yes)	No<br>Yes	Are the outcomes standardized or adjusted for certain factors before model adjustment? For standardized or adjusted outcomes, the standardized or adjusted factors should be included in the adjustment model. If standardized/adjusted outcomes are not used, this criterion is considered to have been met.	0<br>1
**QUALITY OF REPORTING (max=3)**			
Statement of study design (yes)	No<br>Yes	Did the authors present key elements of study design in the paper?	0<br>1
Justification of study design (yes)	No<br>Yes	Did the authors justify the ecologic analysis, the rational and the specific objectives, including any prespecified hypotheses?	0<br>1
Discussion of cross-level bias and limitations (yes)	No<br>Yes	Did the authors caution readers about the limitations of the ecologic design, the ecologic fallacy, the impossibility of extrapolating to a different level?	0<br>1
