# Supplementary material for: Effectiveness of Bacille Calmette-Guerin vaccination policies in reducing infection and mortality of COVID-19: a systematic review
Source: Glob Health Res Policy. 2022 Nov 7;7:42. doi: 10.1186/s41256-022-00275-x (PMC9638327; doi:10.1186/s41256-022-00275-x)
Supplement: Supplementary file 4 — Additional file 4. Critical appraisal of studies using the adapted appraisal tool from Betran et al. and the JBI Critical Appraisal Instruments. [file 41256_2022_275_MOESM4_ESM.pdf]

Obnial et al.
PROSPERO ID: CRD40221244060

CRITICAL APPRAISAL OF ECOLOGICAL STUDIES USING THE ADAPTED APPRAISAL TOOL FROM BETRAN ET AL.

Study	Escobar et al.		Wickramasinghe et al.		Hidvegi and Nichelatti		Szigeti et al.		Sayed et al.		Berg et al.	
Reviewer	1	2	1	2	1	2	1	2	1	2	1	2
Criteria<br>**Study Design**												
Design	2	1	1	2	2	1	1	2	1	2	1	2
Sample Size	0	1	1	1	0	0	0	0	0	0	1	1
Inclusion of units	0	1	1	0	0	0	0	1	0	0	1	1
Data aggregation	3	3	3	1	3	3	3	3	3	3	3	3
Inference	1	1	1	0	1	1	1	1	1	1	1	1
Ecologic Units	1	1	1	1	1	1	1	1	1	1	1	1
Outcome of interest	2	1	1	2	1	1	2	2	1	1	2	1
Source of Data	0	1	1	1	1	1	1	1	1	1	1	1
**Statistical Methodology**												
Analytic Method	1	1	1	1	1	1	1	1	1	1	1	2
Validity of Regression	1	0	1	1	0	0	1	1	0	0	1	1
Use of covariates	2	1	1	1	0	0	1	1	0	0	1	2
Adjustment of covariates	1	1	0	1	0	0	1	1	0	0	1	1
**Quality of Reporting**												
Statement of Design	1	1	1	1	1	1	1	1	1	1	1	0
Justification of Design	1	1	1	1	1	1	1	1	1	1	1	0
Bias and limitations	1	1	1	1	1	1	1	1	1	1	1	1
**TOTAL**	17	16	16	15	13	12	16	18	12	13	18	18
**REVIEW**												
**DECISION**	**INCLUDE**		**INCLUDE**		**EXCLUDE**		**INCLUDE**		**EXCLUDE**		**INCLUDE**	

(Legend: Green – include for that reviewer; Red – exclude for that reviewer)

Obnial et al.
PROSPERO ID: CRD40221244060

Study	Ebina-Shibuya et al.		Abdulah & Hassan		Dolgikh		Klinger et al.	
Reviewer	1	2	1	2	1	2	1	2
Criteria<br>**Study Design**								
Design	2	2	1	1	1	1	1	2
Sample Size	1	1	1	1	0	0	0	0
Inclusion of units	0	1	1	1	0	0	1	0
Data aggregation	3	3	3	3	3	2	3	3
Inference	1	1	1	1	1	1	1	1
Ecologic Units	1	1	1	1	1	1	1	1
Outcome of interest	1	1	1	1	1	1	2	2
Source of Data	1	1	1	1	0	0	1	1
**Statistical Methodology**								
Analytic Method	1	2	1	0	1	1	1	1
Validity of Regression	0	1	1	1	0	0	0	1
Use of covariates	1	2	2	2	0	0	2	2
Adjustment of covariates	1	1	0	1	0	0	1	1
**Quality of Reporting**								
Statement of Design	0	0	1	1	1	1	0	1
Justification of Design	1	0	1	0	1	1	0	1
Bias and limitations	1	1	1	1	0	0	1	1
**TOTAL**	15	18	17	16	10	9	15	18
**REVIEW**								
**DECISION**	**INCLUDE**		**INCLUDE**		**EXCLUDE**		**INCLUDE**	

(Legend: Green – include for that reviewer; Red – exclude for that reviewer)

Obnial et al.
PROSPERO ID: CRD40221244060

Study	Sharma et al.			Hensel et al.			Chimoyi et al.		
Reviewer	1	2	3	1	2	3	1	2	3
Criteria<br>**Study Design**									
Design	2	2	1	1	1	2	1	1	2
Sample Size	0	1	1	1	0	0	0	1	1
Inclusion of units	1	0	0	1	1	0	1	1	1
Data aggregation	3	3	3	3	3	3	3	3	3
Inference	1	1	1	1	1	1	1	1	1
Ecologic Units	0	1	1	0	1	0	1	1	1
Outcome of interest	2	2	2	1	1	2	2	1	0
Source of Data	0	1	1	1	1	1	0	1	1
**Statistical Methodology**									
Analytic Method	1	1	1	1	1	1	1	1	1
Validity of Regression	1	0	0	1	0	1	0	1	0
Use of covariates	1	0	0	1	2	0	0	2	0
Adjustment of covariates	0	0	0	0	1	0	0	1	1
**Quality of Reporting**									
Statement of Design	1	0	0	0	0	1	1	1	1
Justification of Design	1	0	0	1	1	1	1	1	1
Bias and limitations	1	0	0	1	1	1	0	1	1
**TOTAL**	15	12	11	14	15	14	12	18	15
**REVIEW**									
**DECISION**	**EXCLUDE**			**EXCLUDE**			**INCLUDE**		

(Legend: Green – include for that reviewer; Red – exclude for that reviewer)

Obnial et al.
PROSPERO ID: CRD40221244060

Study	Li			Ogimi et al.			Brooks et al.		
Reviewer	1	2	3	1	2	3	1	2	3
Criteria<br>**Study Design**									
Design	1	2	1	2	1	1	2	1	2
Sample Size	0	1	1	1	0	0	1	0	0
Inclusion of units	0	1	1	1	0	0	1	1	0
Data aggregation	3	3	3	3	3	3	3	3	3
Inference	1	1	1	1	1	1	1	1	1
Ecologic Units	0	1	1	1	0	0	1	1	1
Outcome of interest	1	2	2	1	1	2	1	1	2
Source of Data	0	1	1	1	1	1	1	1	1
**Statistical Methodology**									
Analytic Method	1	1	1	2	1	1	2	1	1
Validity of Regression	0	1	2	0	0	1	1	0	1
Use of covariates	0	1	1	2	2	2	2	2	2
Adjustment of covariates	0	1	1	0	1	1	1	0	1
**Quality of Reporting**									
Statement of Design	0	1	1	0	1	1	0	0	1
Justification of Design	0	1	0	0	0	1	0	0	1
Bias and limitations	0	1	1	1	1	1	1	0	1
**TOTAL**	7	19	18	16	13	16	18	12	18
**REVIEW**									
**DECISION**	**INCLUDE**			**INCLUDE**			**INCLUDE**		

(Legend: Green – include for that reviewer; Red – exclude for that reviewer)

Obnial et al.
PROSPERO ID: CRD40221244060

CRITICAL APPRAISAL OF COHORT STUDIES USING THE JBI CHECKLIST FOR COHORT STUDIES

**STUDY**	Chaisemartin & Chaisemartin		Rivas et al.	
**REVIEWER**	1	2	1	2
1. Were the two groups similar and recruited from the same population?	YES	YES	YES	YES
2. Were the exposures measured similarly to assign people to both exposed and unexposed groups?	YES	YES	YES	YES
3. Was the exposure measured in a valid and reliable way?	YES	YES	YES	NO
4. Were confounding factors identified?	NO	YES	YES	YES
5. Were strategies to deal with confounding factors stated?	NO	YES	YES	NO
6. Were the groups/participants free of the outcome at the start of the study (or at the moment of exposure)?	YES	YES	YES	YES
7. Were the outcomes measured in a valid and reliable way?	YES	YES	YES	YES
8. Was the follow up time reported and sufficient to be long enough for outcomes to occur?	YES	YES	NO	YES
9. Was follow up complete, and if not, were the reasons to loss to follow up described and explored?	YES	NO	NO	YES
10. Were strategies to address incomplete follow up utilized?	NO	YES	NO	NO
11. Was appropriate statistical analysis used?	YES	YES	YES	YES
**TOTAL**	8	10	8	8
**REVIEW**				
**DECISION**	**INCLUDE**		**INCLUDE**	

(Legend: Green – include for that reviewer; Red – exclude for that reviewer)
